# Trans/Travesti/+: PrEP in specialized services before and during the COVID-19 pandemic

**DOI:** 10.11606/s1518-8787.2024058005719

**Published:** 2024-10-11

**Authors:** Ramiro Andres Fernandez Unsain, Eliana Miura Zucchi, Lorruan Alves dos Santos, Dulce Ferraz, Paula Massa, Júlia Clara de Pontes, Alexandre Grangeiro, Marcia Thereza Couto

**Affiliations:** I Universidade Católica de Santos. Programa de Pós-Graduação em Saúde Coletiva. Santos, SP, Brasil Universidade Católica de Santos Programa de Pós-Graduação em Saúde Coletiva Santos SP Brasil; II Universidade de São Paulo. Faculdade de Medicina. Grupo de Estudo e Pesquisa em Saúde, Interseccionalidade e Marcadores Sociais da Diferença. São Paulo, SP, Brasil Universidade de São Paulo Faculdade de Medicina Grupo de Estudo e Pesquisa em Saúde, Interseccionalidade e Marcadores Sociais da Diferença São Paulo SP Brasil; III Fundação Oswaldo Cruz. Escola de Governo Fiocruz Brasília. Brasília, DF, Brasil Fundação Oswaldo Cruz Escola de Governo Fiocruz Brasília Brasília DF Brasil; IV Université Lumière Lyon 2. Pôle de Psychologie Sociale. Lyon, France Université Lumière Lyon 2 Pôle de Psychologie Sociale Lyon France; V Universidade de São Paulo. Faculdade de Medicina. Departamento de Medicina Preventiva. São Paulo, SP, Brasil Universidade de São Paulo Faculdade de Medicina Departamento de Medicina Preventiva São Paulo SP Brasil

**Keywords:** Sexual and Gender Minorities, Pre-Exposure Prophylaxis, HIV, Qualitative Research, COVID-19

## Abstract

**OBJECTIVE:**

To understand the perceptions and experiences of transsexual and travesti women and non-binary or gender-fluid people (TGWT+) in health services where they took pre-exposure prophylaxis (PrEP) in the periods before and during the pandemic, focusing on the resignification in the relationship with services and the continuity of HIV prevention via PrEP.

**METHODS:**

Qualitative research and analysis of empirical material generated in the context of broader studies were conducted. A total of 45 semi-structured interviews were conducted with TGWT+ PrEP users in the city of São Paulo and analyzed using iterative thematic content analysis.

**RESULTS:**

The TGWT+ interviewees gave new meanings to their struggles, daily lives and ways of caring for their health and played a central role within them. With its particularities, the COVID-19 pandemic became one more event among all the others that TGWT+ face daily. Alongside the COVID-19 pandemic, the TGWT+ were faced with the HIV epidemic, but with the possibility of prevention via PrEP.

**CONCLUSION:**

Based on a situated and conscious perspective, more explicit in the context of the health crisis, the TGWT+ intertwined conscious actions and care practices in dialog with health services. Further research on these groups from a spatiotemporal dynamic situated in specific contexts of meaning production would make it possible to advance prevention and care strategies, especially in times of health crisis.

## INTRODUCTION

The COVID-19 pandemic had a significant impact on health services and the population^1^. The lack of articulated intersectoral policies and the discontinuity of programs amplified difficulties in accessing programs and services, especially those for HIV prevention^2^.

It is well established that transgender women, *travestis* and non-binary or gender fluid people (TGWT+) have their right to health and access to services disproportionately affected, mainly due to gender-based violence expressed in disrespect for their identities and sexual stigma^3^. That is expressed in the impairment of health care in general and sexual health in particular^4^^), (^^5^.

The adoption of self-care and prevention strategies for HIV and other Sexually Transmitted Infections (STIs) by TGWT+ is mediated by their peer social networks and, at the same time, by the relationship they establish with health professionals and services. Therefore, without intending to make a systematic comparison, it is pertinent to investigate, in the context of the COVID-19 pandemic and before, possible losses and gains related to HIV prevention and the use of pre-exposure prophylaxis (PrEP)^6^.

In the COVID-19 pandemic, especially in Brazil, the possibilities of dealing with exposure to the virus, illness, and its repercussions were mediated by living conditions and access to goods, services and social rights^7^. In this sense, COVID-19 is treated here as an “event”^8^ in the social and political field that has increased the barriers to accessing services on the one hand and, on the other hand, increased the search for healthcare alternatives based on networks and paths that are not necessarily institutional. The proposal by Romano et al.^8^ to consider the pandemic as an event rather than a “disaster” or a “catastrophe” is based on the ideas of Byung-Chul Han^9^, who proposes twisting the hegemonic idea of “epidemic” and starting to describe and explain this reality from the different subjects who are, in fact, the protagonists of the stories surrounding it.

Considering perceptions and experiences from before and during the pandemic, we will analyze the relationships that TGWT+ has established with the services where they receive PrEP. We will explore the possible reframing of these services and look into the continuity of HIV prevention. The proposal is to deepen research that reflects on TGWT+ from a space-time dynamic situated in specific contexts, to rethink prevention and care strategies, especially in times of crisis.

## METHODS

The empirical material analyzed was produced in *PrEP 1519*^10^, *Combina!,*^11^ and *COBra*^12^ studies. *PrEP 1519* and *Combina!* are studies that demonstrate the effectiveness of daily PrEP. *PrEP 1519* was conducted with adolescent cisgender men who have sex with men (MSM) and TGWT+, and *Combina!* was conducted with adults. *COBra*, using a mixed methodology (qualitative and quantitative), assessed the impact of the COVID-19 pandemic on the sexual and mental health of PrEP users who were being followed up in the two studies above.

This article analyzed 45 semi-structured interviews with TGWT+ PrEP users in São Paulo city. Recruitment ensured sample diversification by race/color, schooling, and socioeconomic status (income, occupation, and places of residence, and work). The interviews were carried out by trained researchers in health services from 2018 to 2019, and then via videoconference in the first half of 2021, due to the COVID-19 pandemic.

There were two interview scripts: before and during the COVID-19 pandemic. Although different, the two scripts questioned the interviewees’ relationship with the services and sought to understand their feelings, worries, and concerns about starting and adhering to PrEP, as well as the positive aspects of prophylaxis, such as the ideas of protection, safety, and peace of mind. The interviews conducted during the pandemic explored the impact of COVID-19 on the participants’ living conditions and their continued use of PrEP.

When using iterative thematic inquiry^13^, the aim was to understand the symbolic and material universe of the interviewees and to grasp their perceptions of the relationships they established with the services before and during the pandemic. After examining the content of the interviews, a systematic analysis of the units of meaning selected from the speeches was carried out, assessing their contributions to the overall analytical corpus^14^. The statements were then linked to the analysis themes, ultimately establishing a dialog between the material analyzed, the literature on the subject, and the theoretical framework. In this sense, fictitious names were used to ensure the anonymity of the participants.

It is important to emphasize that the intention was not to compare the answers given before and during COVID-19. In addition, the participants’ perspectives, who constructed their narratives based on life events in time and space, were taken as a reference. In this way, the categories transgender woman, travesti, and non-binary or gender-fluid person comprise historical, social, economic, symbolic, and political dimensions that transcend conjunctures.

The term “transgender women” is commonly used in the USA and other countries to refer to women who were assigned the male sex at birth^15^. In Brazil and some other Latin American countries, however, the terms “travesti(s)” or “trans women” are also used and re-signified by activist groups and communities^16^^), (^^17^^), (^^18^. For this reason, the term “travesti” was used as an identity and political category from an emic perspective. People who identify as non-binary or gender fluid do not use the binary terms male and female to define themselves and may identify themselves as trans^17^.

Lastly, to better understand the two historical moments and examine similarities or differences, the data obtained were broken down into thematic axes: a) routines, b) reception/restraint/confidence in services, and c) time/space dimension.

Based on the work of Romano^8^ and Han^9^, these axes were investigated in terms of the variations in the symbolic and material construction of services and whether they were given new meanings as a result of COVID-19.

The studies *Combina!*, *PrEP 1519,* and *COBra* were approved by the Research Ethics Committee of Hospital das Clínicas and Faculdade de Medicina da Universidade de São Paulo, with opinions No. 2.131.668, No. 3.082.360 and No. 3.988.973, respectively.

All participants were informed about the study’s objectives and their rights regarding participation, as well as their rights in relation to participation, and signed an Informed Consent Form. Parental consent was waived once judicial authorization was granted, in order to preserve the person’s right to participate.

## RESULTS

### Characterization of TGWT+

Most interviewees defined themselves as trans women (41), white (22) or mixed-race (15), heterosexual (38), and aged 15 to 52 (Chart 1), and the age distribution was similar between those interviewed before and during the pandemic.

**Chart 1:** Trans/Travesti/+ women’s data.

N=45	Pseudonym	Age (years)	Skin color/ethnicity	Gender identity	Sexual orientation	Schooling level	Occupation/Job	Housing
Before the COVID-19 pandemic n=18	Cecília	21	White	Trans woman	Heterosexual	Complete primary education	Sex work and online stripping	Family
	Ana Laura	21	White	Trans woman	Heterosexual	Incomplete primary education	Sex work	Friends
	Catarina	41	White	Trans woman	Heterosexual	Complete tertiary education	Nurse, craft making and sex work	Alone
	Olívia	23	Mixed-race	Trans woman	Heterosexual	Incomplete secondary education	Sex work	Alone
	Agatha	41	Mixed-race	Trans woman	Heterosexual	Not mentioned	Social Security (INSS) disability pension Makeup artist	Alone
	Mirella	20	White	Trans woman	Heterosexual	Incomplete secondary education (YAE in progress)	Receiving social benefits	Family
	Sophie	25	Black	Trans woman	Bisexual	Incomplete secondary education	Sex work	Alone
	Stella	37	White	Trans woman	Bisexual	Complete secondary education	Sex work	Alone
	Stefany	25	White	Trans woman	Heterosexual	Complete secondary education	Beauty, sales and sex work	Alone
	Pietra	27	Mixed-race	Trans woman	Heterosexual	Complete secondary education	Sex work	Friends
	Milena	33	White	Trans woman	Heterosexual	Complete secondary education	Sex work and student	Alone
	Marcela	40	White	Trans woman	Heterosexual	Complete tertiary education	Sex work	Alone
	Laís	32	White	Travesti	Heterosexual	Incomplete primary education	Sex work	Alone
	Valentina	20	Mixed-race	Trans woman	Heterosexual	Complete secondary education	Sex work and some clothing sales	Alone
	Janaína	19	White	Trans woman	Pansexual	Complete secondary education	Unemployed	Family
	Dandara	19	Indigenous	Trans woman	Heterosexual	Incomplete secondary education	Sex work, acting and makeup artist	Alone
	Gildete	19	White	Trans woman	Heterosexual	Complete secondary education	Sex work	Alone
	Natália	31	Mixed-race	Trans woman	Heterosexual	Complete secondary education	Sex work	Friends
During the COVID-19 pandemic n=27	Isabel	40	White	Trans woman	Heterosexual	Complete secondary education	Fashion student	Alone
	Bárbara	22	Mixed-race	Trans woman	Heterosexual	Complete secondary education	Dentristy student and sex work	Alone
	Iara	20	White	Travesti	Bisexual/Asexual	Complete secondary education	Unemployed Savings	Boyfriend
	Felícia	19	Mixed-race	Trans woman	Lesbian	Complete secondary education	Cafeteria employee	With friends in a reception center
	Marlucia	18	Mixed-race	Gender-Fluid	Pansexual	Complete secondary education	Unemployed	Family
	Sara	18	White	Trans woman	Heterosexual	Incomplete primary education	Sex work	Family
	Kamily	40	Mixed-race	Trans woman	Heterosexual	Incomplete secondary education	Receptionist	Alone
	Elisa	32	White	Trans woman	Heterosexual	Complete tertiary education	Sex work and hairdressing	Alone
	Mariane	29	Mixed-race	Trans woman	Heterosexual	Incomplete secondary education	Sex work	Friends
	Luna	43	White	Trans woman	Heterosexual	Complete tertiary education	Nurse	Alone
During the COVID-19 pandemic n=27	Eloá	28	Black	Trans woman	Heterosexual	Incomplete secondary education	Sex work	Alone
	Rayssa	28	White	Trans woman	Heterosexual	Complete secondary education	Sex work	Alone
	Alana	32	Mixed-race	Trans woman	Heterosexual	Complete secondary education FMU scholarship student	Sex work	Friend
	Sophia	31	Mixed-race	Trans woman	Heterosexual	Incomplete primary education	Sex work	Alone
	Lívia	28	White	Trans woman	Bisexual	Complete secondary education	Sex work	Alone
	Brenda	35	Black	Trans woman	Heterosexual	Complete secondary education	Sex work and real estate services	Alone
	Evelyn	37	White	Trans woman	Heterosexual	Complete secondary education	Sex work	Alone
	Debora	32	White	Trans woman	Heterosexual	Complete secondary education	Sex work	Alone
	Maitê	19	Mixed-race	Trans woman	Heterosexual	Complete secondary education	Sex work	Alone
	Ana	19	White	Trans woman	Heterosexual	Complete secondary education	Student	Family
	Nina	21	Mixed-race	Trans woman	Heterosexual	Complete secondary education	Makeup artist	Family
	Raquel	52	Indigenous	Trans woman	Heterosexual	Complete secondary education	Hairdressing	Alone
	Jeniffer	18	White	Trans woman	Heterosexual	Complete secondary education	Student	Family
	Betina	21	Black	Trans woman	Heterosexual	Incomplete secondary education	Sex work and nail design	Family
	Mariah	15	Black	Non-binary trans person	Bisexual	Incomplete secondary education	Employee	Family
	Caroline	27	Indigenous	Trans woman	Heterosexual	Complete secondary education	Sex work	Alone
	Joana	43	Mixed-race	Trans woman	Heterosexual	Complete primary education	Acting, makeup artist and sex work	Family

Categories included more recently in LGBTQIAPN+ groups, such as “pansexual”, “asexual”, and “non-binary”, were mentioned by the younger participants; one defined themselves as *travesti*, one as gender-fluid, and another as a non-binary trans person.

Sex work was mentioned by 31 participants, some of whom said they also worked with make-up or hairdressing sometimes. Five participants stated that they had once been sex workers but at the time had other jobs or were beneficiaries of government programs/social benefits. They did not rule out the possibility of returning to sex work if necessary. Nine interviewees said they had never engaged in sex work, all of whom were younger (aged 15 to 21).

Table 1 shows other characteristics, such as housing and schooling.

### The COVID-19 event and the changes in routines

As sex work is one of the ways to elucidate the construction of the world of most of the TGWT+ interviewed, it was proven that the pandemic changed the routine of those who engaged in it. The routine also changed for those who did not, although the impact on daily life was less arduous. At the beginning of the pandemic work activities were very restricted in the first three months,. In a later period, sex work, like so many other activities, took on a new meaning: virtual platforms began to be exploited in the face of a shortage of street customers and the temporary closure of private clubs.

In this context of readaptation, the prevention of HIV and other STIs was no longer central. The COVID-19 event took center stage in terms of protection and prevention.

The COVID-19 pandemic also redefined the role of PrEP professionals and services: as per the interviewees, they were no longer confined to sexual health, but also encompassed the prevention and care of the new health emergency. Moreover, the service and the health professionals were revalued in the context of the health crisis because they organized themselves to continue offering HIV prevention and PrEP.

### Hospitality and trust in the service that goes beyond HIV prevention

All the interviewees acknowledged that the services where they were monitored for PrEP before and during the pandemic were places of hospitality, safety, and trust, where they could talk about different aspects of their lives “almost” without judgment. “Almost” because, for the interviewees, judgment related to sex work or being TGWT+ remained a subtle but insidious symbolic and material barrier with the potential to hinder PrEP adherence. Cecília (Pre-Pandemic/Trans Woman) understood these signs with eloquence: “I did not experience prejudice... [but] they [health professionals] are always surprised by who I am”, she mentioned, referring to the fact that she belongs to the TGWT+ group.

TGWT+ people have health demands that go beyond sexual health and predate COVID-19, and these include procedures associated with gender transition. In this sense, these health services not only provide HIV prevention via PrEP but also include, for example-and in some cases-hormone therapy.

Before the pandemic, the interviewees gave positive reports about the services and their professionals. Olívia (Pre-Pandemic/Trans Woman) commented that she felt welcomed for the first time in a health service: “Oh, I felt... normal. I was very well welcomed”. The mention of feeling “normal” echoes a desire Olívia had held ever since she decided to leave home to “live life as a woman”.

Sophie said she felt welcomed and pointed out that this story of hospitality came from her mother, who was living with HIV:

I had already heard about the service [...], that my mother was HIV positive and... I had already heard about this drug [PrEP]. And when I was here with the doctor... the staff, a nurse, approached me and... welcomed me (Sophie/Pre-Pandemic/Trans Woman).

Stella spoke about how she was welcomed when she thought she had been exposed to HIV and arrived “desperate” at the service:

I got here... I was out of my mind, deranged, and blind (laughs). Everyone was trying to calm me down, and I kept on crying. Then, the infectious disease doctor sat down and spoke with me. “You’re going to take such and such medication. We will give you medication for everything right away. Injection against Syphilis, against Gonorrhea..”. (Stella/Pre-Pandemic/Trans Woman).

Mentions of hospitality and trust in PrEP services remain firm in the statements of those interviewed during the pandemic. However, the COVID-19 event brought out new meanings among participants: some began to question whether PrEP “can be used for other diseases”, in the sense of antiretroviral use aimed at prevention. Alana (During the Pandemic/Trans Woman), for example, assured that she always thanks the service for providing her with an “antiviral” pill that “certainly frees” her from COVID-19. Thus, the crisis caused by the pandemic strained conceptions about communicable diseases, their prevention, treatment, and scope.

Lívia (During the Pandemic/Trans Woman), going further, ensured that: “Because I take PrEP, I think that if a person has a disease and I have sex with them without a condom, maybe I am passing on the cure to them as if it were a vaccine”. According to the interviewees, all these queries and assumptions are listened to and clarified carefully by the health professionals.

Although they expressed trust in and acceptance of the services, some TGWT+ did not exempt certain health professionals from criticism related to severe and systematic scrutiny of their lives, including procedures related to care by health professionals, such as having to answer a questionnaire every three months. However, these complaints do not disqualify the image that the services are protected institutional areas.

Among some TGWT+, the difference in treatment in public and private health services is striking. Two of them, one before and one during COVID-19 admitted that they had sought private health services and had been mistreated, being called by their male names. They also reported aggressive attitudes from health workers, doctors, or nurses. Although they recognized that these episodes could happen in the public sphere, the interviewees reinforced that these incidents in the private sphere were “terrible”, “dangerous”, and “offensive”.

Age is a differentiator in the perception of hospitality. The need for restraint appears to be a complaint among the older interviewees especially among those who lived alone. Among younger women, especially those who live with their families, this need is divided between families and health services. As Marlucia pointed out:

[...] [my mother] has never been as close to me as she is now. However, when I need something related to health [...] I always ask the staff at the VTC [Voluntary Counseling and Testing Centers] for help. If I need a referral [for a medical specialty], I go there and get it (Marlucia/During the Pandemic/Gender-Fluid).

### Temporal/spatial dimension

If one thing was different before and during the pandemic, it was the way in which time and distances were calculated and rearranged. A constant complaint about access to health services before the pandemic had to do with the hours spent commuting.

Milena, a sex worker in the Jardins neighborhood of São Paulo, commented:

It is because it is a bit far here. Since I... live and work in Jardins, for me to come here, I have to get up very early (laughs) (Milena/Pre-Pandemic/Trans Woman).

In the case of Laís, the time spent commuting and at work are potential barriers to adherence to PrEP:

[...] when I go to the service, for my appointments [...] that is the part that bothers me. It is very tiring. I live very far away, right? Then I have to wait, stand in line, right? Waiting to be called (Laís/Pre-Pandemic/*Travesti*).

Pietra reported the same problem and added her dissatisfaction with the organization of the service flow:

Coming to the service for the follow-up... it bothers me... it is too early, in the morning. In reality, it is a lack of organization... Like, because you have to take a test first... then you are called in. It is the clinic [that makes it difficult], not the person who takes care of PrEP, you know? (Pietra/Pre-Pandemic/Trans Woman).

Sometimes, however, the welcoming and attentive professional service made the interviewees decide to spend more time commuting and in more crowded clinics. Carla commented on this:

[...] there is a clinic in Santo André where I used to go, but the service there is completely different. Here I receive a little more attention... the doctor always texts me; you know? But it takes time (Carla/Pre-Pandemic/Trans Woman).

During the pandemic, when it was impossible for users to travel, the service organized “logistics”, in native terms, by which PrEP could reach homes. This included not only PrEP, but also hormone therapies - when prescribed - and HIV self-tests, among other supplies. Another possibility was for people to seek PrEP from the services, which would prepare whatever was necessary for prompt withdrawal.

Although the health services that provide hormone therapy are not necessarily those that provide PrEP, there seems to be a perception that the spaces that offer PrEP, hormone therapy, or other resources are re-signified as a common area of continuity of care. From an interpretative perspective, the health team loses its human form and merges with the place where it works, forming the “service”, a lively area of struggle for a group whose voice is hardly heard.

It is not the purpose of this paper to think about how these logistics were organized but to understand their impact on the lives of TGWT+, considering that complaints of “lost time” due to commuting were frequent before the pandemic.

As reported by Isabel:

During the pandemic I continued to have access to treatment [referring to PrEP], with no problem. I am taking all the medications I used to... Because there [name of the service] when these problems started [COVID-19], they started leaving the prescription ready for us to just go, pick it up and leave. We did not even see a doctor. And there were doctors who called us... and the consultation was like this, via WhatsApp (Isabel/During the Pandemic/Trans Woman).

Luna (During the Pandemic/Trans Woman) commented that appointments went much faster during the pandemic: “I make an appointment at [name of service] with the girl via WhatsApp, and then I see the doctor, and everything goes faster”.

Joana (During the Pandemic/Trans Woman) was blunt about receiving PrEP, both before and during the pandemic: “I am taking PrEP. I have never had any problems getting PrEP”. This comment was common among TGWT+, although there were complaints about hormone therapy in the period before the pandemic. According to Caroline (During the Pandemic/Trans Woman): “This thing, I was supposed to do it, but I did not, you know? I have so many things on my plate, I work and everything... and when I go to this area [where the service is located], I have to spend the whole day there. It is so tiring”.

When talking about commuting, it is also important to consider material issues. Each transport ticket has a cost that is sometimes difficult to cover, especially when several rides are required over short periods. It is worth noting that offering to pay for transportation - a measure adopted in the *PrEP1519* study to mitigate barriers to access - does not necessarily change the perception that going to the service is expensive.

We should also mention that half of the participants interviewed during the pandemic changed the frequency of PrEP use in some way. Not because of a lack of medication but because they reduced or stopped having erotic-sexual encounters. Also, in the case of the *PrEP1519* study, the answers given during the pandemic show that the use of PrEP may have contributed to a programmatic mediation that allowed participants to make autonomous and informed decisions about when or not to use prophylaxis, which is in line with the results of Ferraz and collaborators^19^.

## DISCUSSION

First, it is essential to note that there are no specific references to criticism of public health services or profound changes in their meaning due to the COVID-19 event. Thus, the interviewees reported some changes in the follow-up to PrEP during the pandemic. These changes were positive because they reduced the time spent at the services or the number of visits to them, replacing them with virtual consultations and contacts and dispensing of medication at home. These data, in themselves, establish PrEP services and their professionals as relatively efficient in everyday situations and bulwarks in moments of crisis. One of the reasons for this construction may be that most of health workers who work in services frequented by TGWT+ are trained to provide support and help maintain adherence and bonding.

Although episodes of embarrassment or discomfort occur, the service is mentally mapped^20^ as the space “where PrEP is”, as one of the interviewees assured us. In this sense, PrEP was territorialized^21^ in the pre-pandemic moment as a complex area where the user must converge, and it continued to be territorialized during the pandemic, albeit in reverse, in the sense that the service could be present in the user’s home. Thus, PrEP traveled to the place of residence, reinforcing the bond of care established even before the COVID-19 event. That also happened, according to some interviewees, with other forms of care and treatment, such as hormone therapy, which continued during the crisis. In this way, the pandemic emerged as a facilitator because it promoted, at least conjuncturally, resolutions to the logistics of access to medication.

In general, our results contrast significantly with various studies in which experiences of stigmatization and discrimination in interactions with health professionals were central reasons for difficulties in adhering to and continuing to use PrEP. Disrespect for gender identity^22^^), (^^23^^), (^^24^, ignorance about “transsexuality”,^24^ and representations of trans people as “too sexually active”, implicitly or explicitly^25^, are part of the daily use of PrEP by TGWT+ in various contexts, both in Brazil and in other countries, showing the structural dimension of AIDS stigma^26^ and transphobia that permeates different contexts of the HIV epidemic (concentrated and generalized), health systems (public, private and mixed) and various conditions of wealth distribution and poverty of the population.

Although there were common elements in the period prior to and concomitant with the pandemic, there were also adjustments and strategies to reframe public health services. Thus, along with the understanding that this was a critical moment and that the struggle for survival, in the sense of resisting different forms of structural violence, was still crucial, there was also the need for careful planning in order to avoid possible contact with the COVID-19 virus, which included assessing whether or not to continue doing sex work.

In other words, if the HIV/AIDS epidemic is an event^8^, this type of event is procedural, a permanent and continuous process that is known and can be prevented. In turn, COVID-19, also as an event - and at an early stage - was perceived by the participants as circumstantial and impossible to handle. In this way, it is a temporary event, even if unknown and unpredictable.

In fact, for some participants, the connection between the HIV-AIDS and COVID-19 events materialized in the use of PrEP. As highlighted in the results, the idea that PrEP users could be more protected against coronavirus infection was profusely present. This idea was possibly based on a news broadcast at the beginning of the crisis^27^. Such reports reveal the participants’ perception that sustaining the behavior of HIV prevention could put them at an advantage when facing this unknown and unpredictable event (COVID-19). This aspect was understood similarly by the adolescent and young participants and the adult interviewees.

By experiencing HIV as a continuous event, the TGWT+ took on a conscious logic of using preventive technology to avoid possible exposure to a threatening virus in people who are more vulnerable because they are sex workers. Not only because of this but also because they wanted to be protected from possible exposure to HIV: “It is the only one that has no cure, I need to take it, I cannot forget it”, said Ana (During the pandemic/Trans woman).

Thus, it can be seen that HIV as a social construction is present in the daily lives of TGWT+ PrEP users, with increased protection being the most frequently evoked reason for its use, which is also the case in other studies^22^^), (^^28^.

It is worth noting that the lack of reference to sex work as the only means of earning a living for most of the adolescent/young women interviewed may indicate a generational turning point linked to a more significant presence of LGBTQIAPN+ movements, despite the two conservative governments that had been in power in the previous six years (former presidents Temer, from 2016 to 2018, and Bolsonaro, from 2019 to 2022). However, when the younger women were asked about sex work, transactional sex did not necessarily take on the connotation of work^29^. Although the participants highlighted improvements in the situation of TGWT+ people, they also pointed out the difficulties and structural violence that this population experiences on a daily basis.

When considering distances and commuting to services, it is also important to consider the COVID-19 event as a driver of care models based on telemedicine, perceived by the interviewed TGWT+ as particularly effective. As shown by Ferraz et al.^19^, in dialog with Hoagland et al.^30^, telemedicine implemented in different health services in Brazil has strengthened the relationship of men who have sex with men and TGWT+ with health professionals, as well as adherence to PrEP.

Both before and during the pandemic, the TGWT+ transcended the fortuitous, in the sense of considering COVID-19 as contingent and continued with HIV prevention via PrEP. In this way, the health services and their professionals endured the health crisis of the pandemic together with the TGWT+, an event that, after the initial crisis, was represented as having a lower impact on the possibility of contracting HIV.

The findings and analysis show that the TGWT+ interviewed confronted the COVID-19 pandemic after the initial shock, leading and re-signifying their struggles, daily lives, and ways of taking care of their health.

In this way, the COVID-19 pandemic - without minimizing the importance of the crisis resulting from its emergence and after its most critical phase -became yet another event among all the others they face on a daily basis. Alongside the COVID-19 pandemic, they experience the HIV epidemic, but with the possibility of prevention with PrEP.

The relationship of trust and respect established between the TGWT+ and PrEP services before the pandemic was maintained during the event but reorganized to meet the demands of limiting physical contact and mobility to control COVID-19. In addition to the wisdom of the community and the agency that enabled the participants to adapt, the reorganization of the PrEP service was fundamental to continuing to ensure access to and follow-up of prophylaxis.

TGWT+ does not establish a passive relationship with the PrEP service. Based on a situated and conscious perspective, which is even more explicit at a time of crisis, they interweave thoughtful and practical actions aimed at resistance.
