# Supplementary material for: Trans/Travesti/+: PrEP in specialized services before and during the COVID-19 pandemic
Source: Rev Saude Publica. 2024 Oct 11;58(Suppl 1):12s. doi: 10.11606/s1518-8787.2024058005719 (PMC11573367; doi:10.11606/s1518-8787.2024058005719)
Supplement: Translation

# Translation: Trans/Travesti/+: PrEP em serviços especializados antes e durante a pandemia de covid-19

**Authors:** Ramiro Andres Fernandez Unsain, Eliana Miura Zucchi, Lorruan Alves dos Santos, Dulce Ferraz, Paula Massa, Júlia Clara de Pontes, Alexandre Grangeiro, Marcia Thereza Couto

**Affiliations:** I Universidade Católica de Santos. Programa de Pós-Graduação em Saúde Coletiva. Santos, SP, Brasil Universidade Católica de Santos Programa de Pós-Graduação em Saúde Coletiva Santos SP Brasil; II Universidade de São Paulo. Faculdade de Medicina. Grupo de Estudo e Pesquisa em Saúde, Interseccionalidade e Marcadores Sociais da Diferença. São Paulo, SP, Brasil Universidade de São Paulo Faculdade de Medicina Grupo de Estudo e Pesquisa em Saúde, Interseccionalidade e Marcadores Sociais da Diferença São Paulo SP Brasil; III Fundação Oswaldo Cruz. Escola de Governo Fiocruz. Brasília, DF, Brasil Fundação Oswaldo Cruz Escola de Governo Fiocruz Brasília DF Brasil; IV Université Lumière Lyon 2. Pôle de Psychologie Sociale. Lyon, France Université Lumière Lyon 2 Pôle de Psychologie Sociale Lyon France; V Universidade de São Paulo. Faculdade de Medicina. Departamento de Medicina Preventiva. São Paulo, SP, Brasil Universidade de São Paulo Faculdade de Medicina Departamento de Medicina Preventiva São Paulo SP Brasil

**Keywords:** Minorias sexuais e de gênero, Profilaxia pré-exposição, HIV, Pesquisa qualitativa, Covid-19

## Abstract

**OBJETIVO:**

Compreender as percepções e experiências de mulheres transexuais, travestis e pessoas não binárias ou de gênero fluido (MTrT+) em serviços de saúde que fazem segmento de profilaxia pré-exposição sexual (PrEP), antes e durante o contexto de pandemia, quanto às ressignificações na relação com os serviços e à continuidade da prevenção ao HIV por meio da PrEP.

**MÉTODOS:**

Investigação qualitativa e análise de material empírico produzido no âmbito de estudos mais amplos. Realizaram-se 45 entrevistas semiestruturadas com MTrT+ usuárias da PrEP da cidade de São Paulo, aplicando-se posteriormente a análise de conteúdo temática iterativa.

**RESULTADOS:**

As MTrT+ entrevistadas, com relação à pandemia de covid-19; protagonizam e ressignificam suas próprias lutas, seus cotidianos e suas formas de cuidar da saúde. Com suas particularidades, a pandemia se torna um acontecimento a mais dentre todos os demais que as MTrT+ enfrentam quotidianamente. Paralelamente, transitam pela epidemia de HIV, mas com a possibilidade de se prevenir por meio da PrEP.

**CONCLUSÃO:**

Por uma perspectiva situada e consciente, mais explícita no contexto da crise sanitária, as MTrT+ entrelaçam ações conscientes e práticas de cuidado em diálogo com os serviços de saúde. Aprofundar as investigações sobre esses grupos, a partir de uma dinâmica espaço-temporal situada em contextos específicos de produção de sentido, possibilita avançar em estratégias de prevenção e cuidado, especialmente em momentos de crise sanitárias.

## INTRODUÇÃO

A pandemia de covid-19 impactou sobremaneira os serviços de saúde e a população^1^. A ausência de políticas intersetoriais articuladas e a descontinuidade de programas amplificaram dificuldades de acesso a programas e serviços, sobretudo os de prevenção ao HIV^2^.

Já é bem estabelecido que as mulheres transexuais, travestis e pessoas não binárias ou de gênero fluido (MTrT+) têm seu direito à saúde e acesso aos serviços afetados desproporcionalmente, devido, sobretudo, à violência de gênero que se expressa no desrespeito às identidades e no estigma sexual^3^. Isso se expressa no comprometimento do cuidado ofertado à saúde, em geral, e à saúde sexual, em particular^4^^), (^^5^.

A adoção, por parte das MTrT+, de estratégias de autocuidado e prevenção ao HIV e outras Infecções Sexualmente Transmissíveis (IST) é mediada pelas redes sociais de pares e, de forma concomitante, pela relação que estabelecem com profissionais e serviços de saúde. Portanto, sem pretender realizar uma comparação sistemática, é pertinente investigar de que maneira, no contexto anterior e ao longo da pandemia de covid-19, possíveis prejuízos e ganhos relacionados à prevenção do HIV e ao uso da profilaxia pré-exposição sexual (PrEP)^6^.

Na pandemia de covid-19, especialmente na realidade brasileira, as possibilidades de lidar com a exposição ao vírus, o adoecimento e suas repercussões se mostraram mediadas pelas condições de vida e acesso a bens, serviços e direitos sociais^7^. Nesse sentido, a covid-19 é aqui tratada como um “acontecimento”^8^ no campo social e político que potencializou, por um lado, as barreiras de acesso aos serviços, e, por outro, incrementou a busca por alternativas de cuidado em saúde a partir de redes e percursos não necessariamente institucionais. A proposta de Romano et al.^8^ em considerar a pandemia como um acontecimento, em vez de um “desastre” ou uma “catástrofe”, se apoia nas ideias de Byung-Chul Han^9^, que propõe operar uma torsão na ideia hegemônica de “epidemia” e passar a descrever e explicar essa realidade a partir dos diferentes sujeitos que são, de fato, protagonistas das histórias que circundam esse acontecimento.

Considerando as percepções e experiências antes e durante a pandemia, analisaremos as relações que as MTrT+ estabeleceram com os serviços onde fazem o seguimento da PrEP. Exploraremos possíveis ressignificações desses serviços e indagaremos sobre a continuidade da prevenção ao HIV. A proposta é aprofundar as investigações que refletem sobre as MTrT*+* a partir de uma dinâmica espaço-temporal situada em contextos específicos, com a finalidade de repensar as estratégias de prevenção e cuidado, especialmente em momentos de crise.

## MÉTODOS

O material empírico analisado foi produzido no âmbito dos estudos *PrEP 1519*^10^, *Combina!*^11^ e *COBra*^12^. O *PrEP 1519* e o *Combina!* são estudos demonstrativos da efetividade da PrEP diária, o primeiro em adolescentes homens cisgêneros que fazem sexo com homens (HSH) e MTrT+ e; o segundo, em adultos. O *COBra*, utilizando metodologia mista (qualitativa e quantitativa), avaliou o impacto da pandemia de covid-19 sobre a saúde sexual e mental dos(as) usuários(as) de PrEP em seguimento nos dois estudos citados.

Nesse artigo, foram analisadas 45 entrevistas semiestruturadas realizadas com MTrT+ usuárias da PrEP da cidade de São Paulo. O recrutamento garantiu a diversificação amostral por raça/cor, escolaridade e condição socioeconômica (renda, ocupação e locais de moradia e trabalho). As entrevistas foram realizadas por pesquisadores capacitados nos serviços de saúde, entre 2018 e 2019, e, por videoconferência, no primeiro semestre de 2021, devido à pandemia de covid-19.

Houve dois roteiros de entrevistas: antes e durante a covid-19. Embora diferentes, os dois questionavam a relação das entrevistadas com os serviços e procuraram apreender as sensações, ansiedades e preocupações sobre o início e a adesão à PrEP, além de aspectos positivos da profilaxia, como as ideias de proteção, segurança e tranquilidade. As entrevistas realizadas durante a pandemia exploraram o impacto da covid-19 nas condições de vida das participantes e a continuidade do uso da PrEP.

Empregando a análise temática iterativa^13^, buscou-se compreender o universo simbólico e material das entrevistadas e apreender as percepções das relações que estabelecem com os serviços, antes e durante a pandemia. Após a impregnação dos conteúdos das entrevistas, foi realizada uma análise sistemática das unidades de sentido selecionadas a partir das falas, avaliando seus significados e contribuições para o corpus analítico geral^14^. Em seguida, articulamos as falas com os temas de análise, estabelecendo, finalmente, um diálogo entre o material analisado, a literatura sobre o tema e o referencial teórico. Para assegurar o anonimato das participantes, foram utilizados nomes fictícios.

Ressaltamos que não é nosso intuito comparar o antes e o durante a covid-19. Além disso, tomamos como referência a perspectiva das próprias participantes que constroem suas narrativas por meio de acontecimentos da vida no tempo e espaço. Dessa maneira, as categorias mulher transexual, travesti, pessoa não binária ou fluida compreendem dimensões históricas, sociais, econômicas, simbólicas e políticas que transcendem as conjunturas.

O uso do termo “mulheres transexuais” é comum nos EUA e em outros países para designar mulheres com o sexo masculino atribuído no nascimento^15^. No Brasil, e em alguns outros países latino-americanos, contudo, os termos “travesti(s)” ou “mulheres trans” também são usados e ressignificados pelos coletivos e comunidades^16^^), (^^17^^), (^^18^. Por isso, mantivemos o termo “travesti” como categoria identitária e política numa perspectiva êmica. As pessoas que se identificam como não binárias ou fluidas não utilizam os termos binários masculino e feminino para se definir e podem se identificar como pessoas trans^17^.

Finalmente, para entender melhor os dois momentos históricos e apreciar similitudes ou diferenças, desagregamos os dados obtidos em eixos temáticos: a) rotinas; b) acolhimento/contenção/confiança nos serviços; e c) dimensão temporal/espacial.

A partir das referências de Romano^8^ e Han^9^, percorremos os eixos mencionados pensando nas variações da construção simbólica e material dos serviços, e analisamos se eles foram ressignificados à luz da covid-19.

Os estudos *Combina!*, *PrEP 1519* e *COBra* foram aprovados pelo Comitê de Ética em Pesquisa do Hospital das Clínicas e Faculdade de Medicina da Universidade de São Paulo, com pareceres de nº 2.131.668, nº 3.082.360 e nº 3.988.973, respectivamente.

Todas e todos os participantes foram informadas(os) sobre os objetivos da pesquisa, bem como sobre seus direitos relacionados à participação e assinaram o Termo de Consentimento ou de Assentimento Livre e Esclarecido. O consentimento parental foi dispensado com base em autorização judicial, para preservar o direito de participação da pessoa.

## RESULTADOS

### Caracterização das MTrT+

As entrevistadas, em sua maioria, se definiram como mulheres trans (41) brancas (22) ou pardas (15), heterossexuais (38) e possuíam idade entre 15 e 52 anos (Quadro 1), com equiparação em termos de distribuição etária entre as entrevistadas antes e durante a pandemia.

**Quadro 1:** Dados das Mulheres Trans/Travesti/+

N=45	Pseudônimo	Idade (anos)	Cor/etnia	Identidade de gênero	Orientação sexual	Escolaridade	Ocupação/Trabalho	Moradia
**Antes da pandemia de COVID-19 n=18**	Cecília	21	Branca	Mulher trans	Heterossexual	Ensino fundamental completo	Trabalho sexual e *Online stripper*	Família
	Ana Laura	21	Branca	Mulher trans	Heterossexual	Ensino fundamental incompleto.	Trabalho sexual	Amigas
	Catarina	41	Branca	Mulher trans	Heterossexual	Ensino superior completo	Enfermeira, manufatura de artesanias e trabalho sexual	Sozinha
	Olívia	23	Parda	Mulher trans	Heterossexual	Ensino médio incompleto	Trabalho sexual	Sozinha
	Agatha	41	Parda	Mulher trans	Heterossexual	Não referido	Pensão do Seguro Social (INSS) por incapacidade. Maquiadora	Sozinha
	Mirella	20	Branca	Mulher trans	Heterossexual	Ensino médio incompleto (EJA em curso)	Recebendo benefício social	Família
	Sophie	25	Negra	Mulher trans	Bissexual	Ensino médio incompleto	Trabalho sexual	Sozinha
	Stella	37	Branca	Mulher trans	Bissexual	Ensino médio completo	Trabalho sexual	Sozinha
	Stefany	25	Branca	Mulher trans	Heterossexual	Ensino médio completo	Beleza, vendas e trabalho sexual	Sozinha
	Pietra	27	Parda	Mulher trans	Heterossexual	Ensino médio completo	Trabalho sexual	Amigas
	Milena	33	Branca	Mulher trans	Heterossexual	Ensino médio completo	Trabalho sexual e estudante	Sozinha
	Marcela	40	Branca	Mulher trans	Heterossexual	Ensino superior completo	Trabalho sexual	Sozinha
	Laís	32	Branca	Travesti	Heterossexual	Ensino fundamental incompleto	Trabalho sexual	Sozinha
	Valentina	20	Parda	Mulher trans	Heterossexual	Ensino médio completo	Trabalho sexual/Bicos de venda de roupas	Sozinha
	Janaína	19	Branca	Mulher trans	Pansexual	Ensino médio completo	Desempregada	Família
	Dandara	19	Indígena	Mulher trans	Heterossexual	Ensino médio incompleto	Trabalho sexual/Maquiadora/atriz	Sozinha
	Gildete	19	Branca	Mulher trans	Heterossexual	Ensino médio completo	Trabalho sexual	Sozinha
	Natália	31	Parda	Mulher trans	Heterossexual	Ensino médio completo	Trabalho sexual	Amigas
**Durante a pandemia de COVID-19 n=27**	Isabel	40	Branca	Mulher trans	Heterossexual	Ensino médio completo	Estudante de moda	Sozinha
	Bárbara	22	Parda	Mulher trans	Heterossexual	Ensino médio completo	Estudante de odontologia, Trabalho sexual	Sozinha
	Iara	20	Branca	Travesti	Bissexual/Assexual	Ensino médio completo	Desempregada, Poupança	Namorado
	Felícia	19	Parda	Mulher trans	Lésbica	Ensino médio completo	Funcionária de Lanchonete	Com amigas em centro de acolhimento
**Durante a pandemia de COVID-19 n=27**	Marlucia	18	Parda	Gênero Fluido	Pansexual	Ensino médio completo	Desempregada	Família
	Sara	18	Branca	Mulher trans	Heterossexual	Ensino Fundamental Incompleto	Trabalho sexual	Família
	Kamily	40	Parda	Mulher trans	Heterossexual	Ensino médio incompleto	Recepcionista	Sozinha
	Elisa	32	Branca	Mulher trans	Heterossexual	Ensino superior completo	Trabalho sexual e cabelereira	Sozinha
	Mariane	29	Parda	Mulher trans	Heterossexual	Ensino médio incompleto	Trabalho sexual	Amigas
	Luna	43	Branca	Mulher trans	Heterossexual	Ensino superior completo	Enfermeira	Sozinha
	Eloá	28	Negra	Mulher trans	Heterossexual	Ensino médio incompleto	Trabalho sexual	Sozinha
	Rayssa	28	Branca	Mulher trans	Heterossexual	Ensino médio completo	Trabalho sexual	Sozinha
	Alana	32	Parda	Mulher trans	Heterossexual	Ensino médio completo. Bolsista FMU	Trabalho sexual	Amigo
	Sophia	31	Parda	Mulher trans	Heterossexual	Fundamental incompleto	Trabalho sexual	Sozinha
	Lívia	28	Branca	Mulher trans	Bissexual	Ensino médio completo	Trabalho sexual	Sozinha
	Brenda	35	Negra	Mulher trans	Heterossexual	Ensino médio completo	Trabalho sexual e serviços imobiliários	Sozinha
	Evelyn	37	Branca	Mulher trans	Heterossexual	Ensino médio completo	Trabalho sexual	Sozinha
	Debora	32	Branca	Mulher trans	Heterossexual	Ensino médio completo	Trabalho sexual	Sozinha
	Maitê	19	Parda	Mulher trans	Heterossexual	Ensino médio completo	Trabalho sexual	Sozinha
	Ana	19	Branca	Mulher trans	Heterossexual	Ensino médio completo	Estudante	Família
	Nina	21	Parda	Mulher trans	Heterossexual	Ensino médio completo	Maquiadora	Família
	Raquel	52	Indígena	Mulher trans	Heterossexual	Ensino médio completo	Cabelereira	Sozinha
	Jeniffer	18	Branca	Mulher trans	Heterossexual	Ensino médio completo	Estudante	Família
	Betina	21	Negra	Mulher trans	Heterossexual	Ensino médio incompleto	Trabalho sexual e design de unha	Família
	Mariah	15	Negra	Pessoa trans não binária	Bissexual	Ensino médio incompleto	Funcionária	Família
	Caroline	27	Indígena	Mulher trans	Heterossexual	Ensino médio completo	Trabalho sexual	Sozinha
	Joana	43	Parda	Mulher trans	Heterossexual	Ensino fundamental completo	Atriz, maquiadora, trabalho sexual	Família

Categorias incluídas mais recentemente nos coletivos LGBTQIAPN+, como pansexual ou assexual e não binária, foram referidas pelas mais jovens; uma se definiu como travesti, uma fluida, e outra trans não binária.

O trabalho sexual foi referido por 31 participantes, entre as quais algumas afirmaram realizar outras atividades como “bicos” de maquiagem e cabelereiras. Cinco declararam que já foram trabalhadoras do sexo, mas, no momento, tinham outros trabalhos ou eram beneficiárias de programas governamentais/benefícios sociais. Elas não descartam a possibilidade de voltar ao trabalho sexual, se necessário. Nove declararam jamais ter feito trabalho sexual, todas mais jovens (15 a 21 anos).

Outras características, como moradia e escolaridade, poderão ser visualizadas no Quadro 1.

### O acontecimento covid-19 e as mudanças nas rotinas

Sendo o trabalho sexual uma das maneiras de tornar inteligível a construção do mundo da maioria das MTrT+ entrevistadas, comprova-se que a pandemia modificou a rotina para aquelas que o desempenhavam. Para as que não o desempenhavam, a rotina também foi modificada, embora o impacto na cotidianidade tenha se apresentado como menos árduo. No momento inicial, primeiros três meses, as atividades laborais estiveram muito restritas e, em um segundo momento, o trabalho sexual, como tantas outras atividades, se ressignificou: plataformas virtuais passaram a ser exploradas frente à escassez de clientes de rua e do fechamento temporário de clubes privês.

Nesse contexto de readaptação, a prevenção do HIV e outras ISTs deixa de ser central. O acontecimento covid-19 adquire protagonismo em termos de proteção e prevenção.

O acontecimento covid-19 ressignifica, igualmente, o papel dos e das profissionais e dos serviços de PrEP: ambos passam a ser entendidos como referidos não exclusivamente à saúde sexual, mas expandem suas atribuições, da perspectiva das entrevistadas, para a prevenção e cuidado da nova emergência sanitária. Além disso, o serviço e os/as profissionais de saúde são revalorizados, a partir da conjuntura da crise sanitária, por se organizarem para continuar ofertando a prevenção do HIV e o oferecimento da PrEP.

### Acolhimento e confiança no serviço que extrapola a prevenção ao HIV

Todas as entrevistadas reconhecem que os serviços onde fazem acompanhamento da PrEP, antes e durante a pandemia, são lugares de acolhimento, contenção e confiança, onde elas podem relatar diferentes dimensões da vida “quase” sem julgamentos. “Quase” porque, para as entrevistadas, o julgamento relacionado ao trabalho sexual ou por serem MTrT+ permanece como uma sutil, mas insidiosa, barreira simbólica e material com potencial para prejudicar a adesão à PrEP. Cecília (Pré-Pandemia/Mulher Trans) percebe esses sinais com eloquência: “Não sofri preconceito... [mas] eles [profissionais da saúde] sempre ficam surpresos com quem eu sou”, em referência ao seu pertencimento ao coletivo de MTrT+.

Para além da saúde sexual, há outras demandas de saúde das MTrT+ anteriores à covid-19, incluindo os procedimentos associados à transição de gênero. Nesse sentido, os serviços de saúde não atendem somente a prevenção ao HIV através da PrEP, mas também, por exemplo - e em alguns casos -, as terapias de hormonização.

As entrevistadas, antes da pandemia, explicitam relatos positivos em relação aos serviços e seus profissionais. Olívia (Pré-Pandemia/Mulher Trans) comenta que se sentiu acolhida pela primeira vez em um serviço de saúde: “Ah, eu me senti... normal. Fui muito bem recebida”. Ser “normal” ecoa em Olívia como um desejo antigo, desde que decidiu sair de casa para “viver a vida como mulher”.

Sophie conta que se sentiu acolhida e destaca que essa história de acolhimento vem da mãe, que vivia com HIV:

Eu já tinha ouvido falar do serviço [...] que minha mãe era soropositiva e... eu já tinha ouvido falar desse medicamento [a PrEP]. E quando eu tava aqui com a doutora... o pessoal, uma enfermeira, me abordou e... me acolheu (Sophie/Pré-Pandemia/Mulher Trans).

Stella relata seu acolhimento quando pensou que tinha sido exposta ao HIV e chegou “desesperada” ao serviço:

Eu cheguei aqui... eu tava louca, transtornada e cega (riso). Todo mundo tentando me acalmar, e eu chorava. Aí, depois que o infectologista se sentou, conversou comigo. “Você vai tomar tal medicação. A gente vai já te dar medicação pra tudo. Injeção contra o Sífilis, contra a Gonorreia..”. (Stella/Pré-Pandemia/Mulher Trans).

O acolhimento e a confiança nos serviços de PrEP seguem firmes nas percepções das entrevistadas durante a pandemia. Todavia, o acontecimento covid-19 faz emergir ressignificações entre participantes: algumas passaram a se questionar se a PrEP “serve para outras doenças”, no sentido do uso de antirretroviral visando a prevenção. Alana (Durante a Pandemia/Mulher Trans), por exemplo, assegura que sempre agradece ao serviço por estar tomando um comprimido “antiviral” que, “com certeza” a “livra” da covid-19. Assim, a crise provocada pela pandemia tensiona concepções sobre as doenças transmissíveis, sua prevenção, tratamento e alcances.

Lívia (Durante a Pandemia/Mulher Trans), indo além, assegura: “Como eu tomo PrEP eu penso que, se a pessoa tiver alguma doença e eu transo sem camisinha, quem sabe eu estou passando a cura pra ela, como se fosse uma vacina”. Segundo as interlocutoras, todas essas consultas e suposições são ouvidas e esclarecidas com atenção pelos e pelas profissionais de saúde.

Ainda que explicitem confiança e acolhimento em relação aos serviços, algumas MTrT+ não eximem certos e certas profissionais de saúde de críticas relacionadas a um escrutínio severo e sistemático de suas vidas, inclusive com relação aos procedimentos relacionados ao atendimento por parte dos e das profissionais de saúde, como ter que responder um questionário a cada três meses. Todavia, as queixas e reclamações não chegam a desqualificar a imagem de que os serviços são âmbitos institucionais resguardados.

Chama a atenção, entre algumas MTrT+, a diferença de trato nos serviços de saúde públicos e privados. Duas delas, uma antes e outra durante a covid-19, admitem ter buscado serviços de saúde privados e relatam situações de maltrato, no sentido de serem chamadas pelo nome masculino. Também narram atitudes agressivas dos e das trabalhadores(as) da saúde, sejam eles ou elas médicos(as) ou enfermeiros(as). Embora reconheçam que esses episódios podem acontecer na esfera pública, reforçam os incidentes no privado como “terríveis”, “perigosos” e “ofensivos”.

A idade é um diferencial na percepção de acolhimento. A necessidade de contenção aparece como uma reclamação das entrevistadas mais velhas e, especialmente, entre aquelas que moram sozinhas. Entre as mais jovens, especialmente as que moram com as famílias, essa necessidade aparece se dividindo entre as famílias e os serviços de saúde. Como destaca Marlucia,

[...] [minha mãe] nunca esteve tão próxima quanto agora. Todavia, quando preciso algo relacionado à saúde [...] sempre peço auxílio ao pessoal do CTA [Centro de Testagem e Acolhimento]. Se eu preciso de um encaminhamento [para alguma especialidade médica], eu vou lá e pego (Marlucia/Durante a Pandemia/Gênero Fluido).

### Dimensão temporal/espacial

Se algo foi diferente antes e durante a pandemia, foi a maneira com que o tempo e as distâncias foram calculados e remanejados. Uma queixa constante para o acesso ao serviço de saúde, antes da pandemia, eram as horas despendidas de deslocamento.

Milena, trabalhadora sexual no bairro dos Jardins, em São Paulo, comenta:

É porque aqui é um pouquinho longe. Como eu... moro e atendo no Jardins, pra mim vim pra cá, eu tenho que acordar muito cedo (riso) (Milena/Pré-Pandemia/Mulher Trans).

No caso de Laís, o tempo gasto de deslocamento e no serviço são barreiras potenciais para adesão à PrEP:

[...] na ida ao serviço, pra fazer o acompanhamento [...] essa parte que me incomoda. É muito cansativo. Eu moro muito longe, né? Aí, tem que passar, pegar uma fila, né? Aguardar ser chamada (Laís/Pré-Pandemia/Travesti).

Pietra relata o mesmo problema e acrescenta seu descontentamento em relação à organização do fluxo de atendimento:

A vinda ao serviço pra fazer o acompanhamento... me incomoda... é muito cedo, de manhã. Na verdade, o que é a falta de organização... Tipo, porque cê tem que colher exame antes... aí depois passa. É o posto [quem dificulta], não a pessoa que cuida da PrEP, entendeu? (Pietra/Pré-Pandemia/Mulher Trans).

Mas, por vezes, o atendimento profissional acolhedor e atento faz com que as interlocutoras decidam passar mais tempo no transporte público e em um serviço mais lotado de pacientes. Carla comenta:

[...] lá em Santo André tem um posto aonde eu ia, só que o atendimento de lá é completamente diferente. Aqui eu sinto um pouco mais de atenção... o médico sempre manda mensagem, entendeu? Mas demora (Carla/Pré-Pandemia/Mulher Trans).

Durante a pandemia, perante a impossibilidade de deslocamento das e dos usuárias e usuários, o serviço organizou uma “logística”, em termos nativos, através da qual a PrEP chegava aos domicílios. E não somente a PrEP, mas as terapias de hormonização - quando prescritas - e os autotestes de HIV, entre outros insumos. Outra possibilidade era que as pessoas procurassem a PrEP nos serviços, que preparavam o que fosse necessário para a pronta retirada.

Embora os serviços de saúde que acompanham a hormonização não sejam necessariamente os de PrEP, parece existir uma percepção de que os espaços que oferecem a PrEP, a hormonização ou outros recursos se ressignificam como um âmbito comum de continuidade de cuidado. Sob uma perspectiva interpretativa, a equipe de saúde perde a forma humana e se funde com o recinto onde atua, conformando o “serviço”, área de luta animada de um coletivo cuja voz é dificilmente ouvida.

Não é o intuito deste trabalho pensar a maneira como essa logística foi organizada, mas entender seus impactos na vida das MTrT+, considerando que as queixas de “tempo perdido” com os deslocamentos eram frequentes no período anterior à pandemia.

Assim relata Isabel:

Durante a pandemia continuei tendo acesso ao tratamento [referindo-se à PrEP], sem problema. Eu tô tomando todos os medicamentos que eu tomava... Porque lá [nome do serviço] quando começou esses problemas [covid-19], eles começaram a deixar a receita pronta pra gente só ir lá buscar e já vir embora. Nem passava no médico. E tem médicos que ligavam pra gente... e era consulta assim, pelo WhatsApp (Isabel/Durante a Pandemia/Mulher Trans).

Luna (Durante a Pandemia/Mulher Trans) comenta que o atendimento durante a pandemia foi muito mais rápido: “Eu agendo no [nome do serviço], com a menina pelo WhatsApp, e passo com a médica e aí faço tudo mais rápido”.

Joana (Durante a Pandemia/Mulher Trans) é contundente com relação à dispensação de PrEP, tanto antes como durante a pandemia: “Estou tomando PrEP. Eu nunca tive problemas em obter a PrEP”. Esse comentário é comum entre as MTrT+, embora surjam reclamações com relação à homonização no período prévio à pandemia. Segundo Caroline (Durante a Pandemia/Mulher Trans): “Esse negócio, era pra eu ter feito, mas nem fiz, sabe? É tão corrido pra mim que eu, eu trabalho e tudo... aí quando eu vou pra esse lado [região onde fica o serviço], aí é o dia todo. É tão cansativo”.

É importante, ao falar de deslocamento, se ater também às questões materiais. Cada passagem tem um custo que, por vezes, é difícil de cobrir, especialmente quando são necessárias várias viagens em períodos curtos. Cabe ressaltar que a oferta de valor para custear o transporte - medida adotada no estudo *PrEP1519* para mitigar barreiras de acesso - não necessariamente muda a percepção de que ir ao serviço é oneroso.

Devemos mencionar também que metade das entrevistadas durante a pandemia alterou de alguma maneira a frequência de uso da PrEP. Não por falta de comprimidos, mas porque reduziram ou deixaram de ter encontros erótico-sexuais. Ainda no caso da pesquisa *PrEP1519*, durante a pandemia os resultados demonstram que a utilização da PrEP pode ter contribuído para uma mediação programática que permitiu às participantes decidirem de forma autônoma e informada sobre quando usar ou não a profilaxia, coincidindo com os resultados de Ferraz e colaboradores^19^.

## DISCUSSÃO

Primeiramente, é importante destacar que não aparecem referências especificamente críticas aos serviços públicos de saúde, ou profundas ressignificações, decorrentes do acontecimento covid-19. Destarte, as entrevistadas narraram que houve algumas mudanças no seguimento à PrEP durante a pandemia. As mudanças foram avaliadas como positivas por diminuírem o tempo de permanência no local de atendimento ou reduzirem a quantidade de idas ao serviço de saúde, pela substituição por consultas e contatos virtuais, assim como pelo recebimento de medicação em casa. Esses dados, em si mesmos, constroem os serviços de PrEP e seus e suas profissionais como relativamente eficientes em situações habituais e baluartes em situações de crise. Uma das razões dessa construção pode se basear em que a maioria dos e das trabalhadores e trabalhadoras da saúde que atuam nos serviços que são frequentados por pessoas MTrT+ são capacitados para dar suporte e auxiliar na manutenção da adesão e vinculação.

Embora aconteçam episódios de constrangimento ou desconforto, o serviço é mapeado mentalmente^20^ como o espaço “onde está a PrEP”, como assegura uma das entrevistadas. Nesse sentido, a PrEP está territorializada^21^ no momento pré-pandêmico como um âmbito complexo para onde a usuária deve convergir; e ela continua territorializada durante a pandemia, mesmo que de modo reverso, no sentido de que o serviço pode se fazer presente na casa da usuária. Assim, a PrEP viaja até o lugar de residência reforçando o vínculo de cuidado estabelecido antes mesmo do acontecimento covid-19. O que acontece também, segundo algumas interlocutoras, com outros cuidados e tratamentos, como, por exemplo, a hormonização que continuou durante a crise. Dessa maneira, a pandemia emerge como um facilitador, porque promove, pelo menos conjunturalmente, resoluções para a logística de acesso à medicação.

De modo geral, nossos resultados contrastam sobremaneira com diversos estudos em que as experiências de estigmatização e discriminação na interação com profissionais de saúde constituíam razões centrais para dificuldades de adesão e de continuidade do uso da PrEP. O desrespeito à identidade de gênero^22^^), (^^23^^), (^^24^, o desconhecimento sobre “transexualidade”^24^ e representações de pessoas trans como “demasiadamente ativas sexualmente”, de forma implícita ou explícita^25^, fazem parte do cotidiano de uso da PrEP pelas MTrT+ em diversos contextos, tanto no Brasil como em outros países, mostrando a dimensão estrutural do estigma da Aids^26^ e da transfobia que atravessa diferentes contextos da epidemia de HIV (concentrada e generalizada), sistemas de saúde (universais, privados e mistos) e diversas condições de distribuição de riqueza e pobreza da população.

Embora haja elementos comuns no período anterior e concomitante à pandemia, isso não significa que não tenham ocorrido ajustes e estratégias de ressignificação dos serviços públicos de saúde. Assim, junto com a compreensão de que esse momento constituía uma conjuntura crítica e de que a luta pela sobrevivência, no sentido de resistir às diferentes violências estruturais, seguia sendo crucial, se acrescentou a necessidade de um planejamento criterioso com o intuito de evitar possíveis contatos com o vírus da covid-19, como a avaliação da possibilidade de continuar ou não realizando trabalho sexual.

Em outras palavras, se a epidemia de HIV-aids é um acontecimento^8^, este tipo de acontecimento é processual, um devir permanente e contínuo que é conhecido e pode ser prevenido. Por sua vez, a covid-19, também como acontecimento - e no momento inicial -, foi percebido pelas participantes como conjuntural e impossível de manejar. Dessa maneira, é um acontecimento passageiro, ainda que desconhecido e imprevisível.

De fato, para algumas participantes, a conexão entre os acontecimentos HIV-aids e covid-19 se materializa através do uso da PrEP. Como destacado nos resultados, a ideia de que usuárias de PrEP poderiam estar mais protegidas contra a infecção pelo coronavírus se faz presente profusamente. Ideias possivelmente embasadas em notícias veiculadas naquele momento do início da crise^27^. Tais relatos demonstram a percepção de que sustentar o comportamento de se prevenir do HIV poderia lhes colocar em lugar de vantagem para enfrentar o acontecimento desconhecido e imprevisível (covid-19). Este aspecto é compreendido de maneira similar tanto nas participantes adolescentes e jovens como nas entrevistadas adultas.

Ao vivenciarem o HIV como um acontecimento contínuo, as MTrT+ se apropriam de uma lógica consciente de uso de tecnologia preventiva para evitar uma possível exposição a um vírus ameaçador em pessoas em situação de maior vulnerabilidade, por serem trabalhadoras sexuais. Mas não somente por isso; por desejarem estar protegidas perante uma possível exposição ao HIV: “É a única que não tem cura, preciso tomar, não posso esquecer”, assevera Ana (Durante a pandemia/Mulher Trans).

Dessa forma, o HIV como construção social está presente no dia a dia das MTrT+ usuárias de PrEP, sendo o aumento da proteção a razão mais frequentemente evocada para seu uso, o que também se verifica em outras pesquisas^22^^), (^^28^.

Vale destacar que a não referência ao trabalho sexual como a única possibilidade de sustento, para a maioria das entrevistadas adolescentes/jovens, pode indicar um ponto de inflexão geracional atrelado a uma maior presença de movimentos LGBTQIAPN+, apesar de terem atravessado dois governos conservadores nos últimos seis anos (ex-presidentes Temer, de 2016 a 2018, e Bolsonaro, de 2019 a 2022). Todavia, quando interrogadas sobre trabalho sexual, o sexo transacional não assumiu necessariamente, na faixa etária das mais jovens, uma conotação de trabalho^29^. Embora as participantes salientem melhoras na situação das pessoas MTrT+, também apontam as dificuldades e as violências estruturais que esta população atravessa no dia a dia.

Pensando as distâncias e os deslocamentos até os serviços, é importante também considerar o acontecimento covid-19 como impulsionador de modelos de atenção baseados na telemedicina, percebidos pelas MTrT+ como particularmente eficazes. De fato, como Ferraz et al.^19^ demonstram, em diálogo com Hoagland et al.^30^, a telemedicina implementada no Brasil em diferentes serviços de saúde fortaleceu a relação entre homens que fazem sexo com homens e MTrT+ com profissionais da saúde, assim como a adesão à PrEP.

As MTrT+, tanto antes como durante a pandemia, transcenderam o fortuito, no sentido de considerar a covid-19 como contingente e continuaram com a prevenção ao HIV por meio da PrEP. Assim, os serviços de saúde, e seus e suas profissionais, atravessaram junto a elas a crise sanitária da pandemia, acontecimento que, depois da crise inicial, foi representado com um peso menor do que a possibilidade de contrair o HIV.

Os achados e as análises apontam que as MTrT+ entrevistadas, depois do sobressalto inicial, enfrentaram a pandemia de covid-19, protagonizando e ressignificando suas próprias lutas, seus cotidianos e suas formas de cuidar da saúde.

Assim, a pandemia de covid-19 - sem minimizar a importância da crise decorrente da sua emergência e passada a sua fase mais crítica - se torna um acontecimento a mais entre todos os outros que enfrentam na quotidianidade. Paralelamente à pandemia de covid-19, transitam pela epidemia de HIV, mas com a possibilidade de se prevenir através da PrEP.

A relação de confiança e respeito estabelecida entre as MTrT+ e os serviços de PrEP antes da pandemia foi mantida, todavia reorganizada, para responder às exigências de limitação dos contatos físicos e da mobilidade para controle da covid-19. Além da sabedoria comunitária e da agência que permitiu às participantes esta adaptação, a reorganização do serviço de PrEP foi fundamental para continuar assegurando acesso e seguimento da profilaxia.

As MTrT+ não constroem uma relação passiva com o serviço de PrEP. Por meio de uma perspectiva situada e consciente, ainda mais explícita em uma conjuntura de crise, entrelaçam ações ponderadas e práticas, cujo objetivo é resistir.
